# The role of mother’s knowledge and attitudes in shaping childcare in a developing country: A cross-sectional study

**DOI:** 10.1371/journal.pgph.0006824

**Published:** 2026-08-17

**Authors:** José Nelson Maria Vidigal, Lely Lusmilasari, Sri Hartini

**Affiliations:** 1 Department of Nursing, Faculty of Health Sciences, UNIKA Indonesia Santu Paulus Ruteng, Manggarai, NTT, Indonesia; 2 Department of Pediatric and Maternity Nursing, Faculty of Medicine, Public Health and Nursing, Gadjah Mada University, Yogyakarta, Indonesia; Al-Bayan University, IRAQ

## Abstract

This study aims to analyze the relationship between maternal knowledge and attitudes and maternal behavior in caring for stunted children aged 24–35 months. Additionally, it examines how demographic variables are related to these independent variables. A cross-sectional study design was employed, focusing on mothers of stunted children aged 24–35 months in an area of a developing country (n = 56). We utilized a modified version of a previous knowledge and attitude assessment tool. The instrument measuring maternal behavior in caring for stunted children remained unmodified. The study commenced in April 2025, following the acquisition of ethical approval. Data collection included demographic questionnaires, respondent identity forms, informed consent, knowledge assessments, attitude surveys, and evaluations of maternal behavior regarding the care of stunted children. The analysis revealed no significant relationship between maternal knowledge and behavior in caring for stunted children (p = 0.268; p > 0.05). However, there was a significant positive correlation between maternal attitudes and maternal behavior (p = 0.000; p < 0.05). As maternal knowledge decreases, the behavior of caring for stunted children tends to improve. Conversely, an increase in knowledge correlates with a decrease in caring behavior. On the other hand, an increase in positive attitudes towards caring for stunted children leads to an improvement in maternal behavior, while a decrease in attitudes results in poorer care behavior. Behaviors related to caring for stunting include four essential domains, which can serve as a framework for independent assessments of mothers caring for stunted children aged 24–35 months.

## Introduction

Stunting is one of the health problems that often experienced by toddlers every year in developing countries [1]. Knowledge about stunting plays a role in the attitude and behavior of maternal care for stunted children aged 24–35 months. Conversely, a lack of maternal expertise is also a factor that can influence maternal care behavior for their toddlers [2,3]. Related to the problem of stunting, data shows that in an area of a developing country, there are several factors that are significantly related to the incidence of stunting in children aged 12–60 months. Some of these factors are exclusive breastfeeding, history of immunization, history of diarrhea, and loWae Mbelengaternal knowledge about nutrition (78%) [4].

Globally, an estimated 148.1 million children under the age of 5 were stunted in 2022. In the Southeast Asia region, there are 49.8 million children under the age of 5 who are stunted [World Health Organization/ 5]. Stunting is considered a chronic problem if the prevalence rate in a country is more than 20% [6]. Several countries in Southeast Asia that have stunting rates above 20% are Indonesia (21.6%) Setiyawati, et al. [7], Timor Leste (45.1%), Cambodia (22.3%), Laos (27.7%), Malaysia (21.9%), Myanmar (24.1%), and the Philippines (28.8%) [5,8]. Although the prevalence of stunting in a developing country has decreased from 24% in 2021 to 19.8% in 2024, the decrease has not reached the expected target in 2024, which is 14% [9].

Nationally, data shows that toddlers (0–59 months) in a Province of developing country suffer the most from severe stunting (11.7%) and stunting (26.2%). The most common age classification is 24–35 months, with a presentation of severe stunting of 6.9% and stunting of 18.8% [10]. One of the districts in a Province of developing country that has a high stunting rate is Manggarai. In the period of August 2023, the number of stunted/short toddlers (aged 0–59 months) was 3480 (13.1%) spread across 12 sub-districts in Manggarai Regency. One of the Health Centers with the highest percentage of stunting is the Wae Mbeleng Health Center (Ruteng Sub District) with 315 stunted children (27.49%) [11].

Care behavior is an activity carried out by mothers in caring for toddlers by providing food, psychosocial stimulation for children, practicing cleanliness and sanitation of the child’s environment, and seeking treatment when the child is sick [12]. Data shows that the majority of mothers behave poorly in providing food (83.5%) [13]. Other studies show that half (50%) of the mothers studied in Australia and India (Mumbai) tend to use unresponsive feeding practices, food restrictions, pressure to eat, and passive feeding in toddlers [14]. Several other studies show that maternal activity in providing stimulation to toddlers is still lacking. Poor or inadequate stimulation can have an impact on inappropriate child development and a greater risk of stunting in children [15].

Knowledge is an area that has an important influence on being able to create a person’s behavior [16]. Sufficient maternal knowledge about stunting since pregnancy is expected to be able to increase positive maternal care attitudes and behaviors in caring for their children [17]. However, in reality, other studies have found that care behavior in the form of providing nutrition from mothers to stunted children is still lacking due to lack of knowledge. Mothers with poor knowledge have a 1.644 times greater risk of having stunted toddlers compared to mothers with good knowledge [18]. Lack of good knowledge and awareness about stunting care presents a serious challenge to changing behavior related to stunting care [19].

Another factor related to maternal care for stunted children is attitude. Attitude is a response that is still closed to a stimulus or object. The manifestation of attitudes cannot be seen, but can only be interpreted [20]. Positive attitudes and behaviors can be formed from good knowledge [18]. A positive maternal attitude regarding the care of stunted children is manifested in a high curiosity in obtaining information about the care of stunted children. One of them is through choosing the right food [21].

One of the current maternal care actions is feeding stunted children. If this care is not carried out, it will have a negative impact. The short-term impacts are morbidity and mortality in toddlers, the medium term is related to low intellectuality, cognitive abilities, academic achievement, and physical performance, and the long term is related to the quality of human resources and degenerative disease problems in adulthood. In addition, stunted children have a higher risk of experiencing developmental delays compared to children who are not stunted [22–24]. If during the growth and development period the child does not receive immediate treatment, it will have an irreversible or permanent negative impact on his future life [25].

Currently, maternal care for stunted children focuses on the 8 pillars of Community-Based Total Sanitation (STBM-stunting). The eight pillars are: Stop Defecating in the Open (SBS), Washing Hands with Soap (CTPS), Management of Drinking Water and Food in Households (PAMM-RT), Household Waste Security (PS-RT), Household Liquid Waste Security (PLC-RT), fulfillment of nutrition for pregnant women, Infant and Child Feeding (PMBA), and growth monitoring (Ministry of Health of the a developing country, 2018). Although many studies have linked knowledge and attitude variables with stunting incidence, stunting prevention, and various strategies to encourage stunting reduction, there are not many that link knowledge and attitudes with maternal behavior in caring for stunted children, especially in an inland district of a developing country.

The main objective of this paper is to analyze the relationship between maternal knowledge and attitudes with maternal behavior in caring for stunted children aged 24–35 months. The second objective is to determine the relationship between maternal education, maternal age, maternal occupation, family income, provision of health education by health workers with maternal behavior in caring for stunted children aged 24–35 months.

## Materials and methods

### Study design

Recruitment for this analytical observational study with a cross-sectional design began on 21^st^ April 2025, and ended on 26^th^ April 2025.

### Sampling methodology

The accessible population in this study were mothers with stunted and severely stunted toddlers. The population was determined from data from the Wae Mbeleng Health Center, Manggarai Regency in March 2025, totaling 173 toddlers. The number of samples in this study was 56 mothers with stunted and severely stunted children aged 24–35 months at the Wae Mbeleng Health Center. The inclusion criteria for this study were: Mothers with stunted and severely stunted children aged 24–35 months at the Wae Mbeleng Health Center, mothers who could read and write based on respondents’ reports, mothers who had lived for 5 consecutive years in Wae Mbeleng and were not nomadic based on reports from respondents and local health workers, mothers who were willing to be respondents in this study. Meanwhile, the exclusion criteria are mothers who work as health workers/cadres.

### Variabels

1. Dependent variable: Maternal behavior in caring for stunted children aged 24–35 months.
2. Independent variables: Maternal knowledge and maternal attitudes in caring for stunted children aged 24–35 months.
3. Demographic variables: Maternal education, maternal age, maternal occupation, family income, provision of health education from health workers.

### Data collection tools

The data collection instruments in this study used demographic data instruments, informed consent sheets, maternal knowledge instruments, maternal attitude instruments and stunted childcare behavior instruments [26]. Of the three main instruments, the knowledge and attitude instruments have been modified according to the purpose of the writing. The modification involved three validators who were experts in nutrition, stunting researchers, and the community. The instrument validation carried out was content validity and construct validity. The results of the validity test on the knowledge and attitude questionnaires each showed that all statement items had an Aiken coefficient (V) of 0.77-1.00. This means that all statement items have moderate to high validity.

In addition to conducting Aiken’s V analysis, researchers have also conducted content validity tests using the Content Validity Index (CVI) and Content Validity Ratio (CVR) methods on two questionnaires, namely the knowledge and attitude questionnaires of mothers in caring for stunted children aged 24–35 months. After receiving input from the three experts, the researchers made revisions and the results of the overall test of the questionnaire items were declared valid (1). This shows that this instrument is valid and can be used to measure the variables studied. Based on the results of the content validity assessment, it can be concluded that both questionnaires (knowledge and attitude) can be developed and used in this study. The construct validity of the knowledge and attitude questionnaire was conducted on 30 respondents of mothers with stunted children aged 24–35 months at a community health center, in an inland province of a developing country. The reason for conducting the validity test at the Health Center was because it involved respondents who had similar characteristics, an area that was quite close to the research location, and a stunting research sample that was almost the same in number, namely 22.68% [11].

The construct validity test inthis study was analyzed using the Pearson Product Moment correlation formulation which aims to see the correlation between the scores of each statement item and the total score. After completing the content and construct validity tests, the researcher also conducted a reliability test of the two questionnaires. The reliability test on the knowledge questionnaire used Kuder Richardson (KR-20) with KR-20 result criteria ranging from 0 to 1. The closer to 1, the higher the reliability of the test [27]. Based on the results of the questionnaire test of mothers’ knowledge in caring for stunted children aged 24–35 months, the KR-20 value obtained was 0.637, which means the degree of reliability is high. Meanwhile, the results of the attitude questionnaire test used Cronbach Alpha with reliable criteria > 0.70 for the standard error rate (5% in 30 respondents), while < 0.70 was declared unreliable [28]. All questionnaires tested in this study were in the high to moderate reliability category (0.893 > 0.70), so all questionnaires in this study were reliable for use in this study.

### Ethical approval statement

i) *The name of the Institutional Review Board or Ethics Committee*

Before starting the research, ethical clearance was submitted to the Medical and Health Research Ethics Committee (MHREC) Faculty of Medicine, Public Health and Nursing Gadjah Mada University.

ii) *The approval number, or a statement that approval was granted by the named board*

The ethical clearance was issued on April 17, 2025 with the number: KE/FK/0613/EC/2025.

iii) *A statement that formal consent was obtained in written*

Before the informed consent process, prospective respondents (mothers with stunted children) received written explanation about voluntary participation in the study, research procedures, research subject obligations, risks and side effects, benefits, confidentiality, compensation, funding, and other additional information., as outlined on the prospective respondent explanation sheet. They agreed to the recruitment, they provided personal information and information about their children accurately, signed the informed consent form, and proceeded to complete the written questionnaire. All data provided will be kept confidential and used solely for research purposes.

### Data collection

In quantitative research, there are several data collection methods, including questionnaires, and observations, and interviews [29]. Data collection in this study used a questionnaire technique (self-report) which was conducted from 21^st^ April 2025, and ended on 26^th^ April 2025. This technique was carried out by giving a set of written statements to respondents to be answered at the Integrated Health Post location. Data were then collected from each integrated health post after the activities were completed and compiled at the Wae Mbeleng Health Center. Because the Wae Mbeleng Health Center has 26 integrated health posts spread across 7 villages, every day after completing the research and collecting the questionnaire sheets at the integrated health post location, the researcher immediately returned to the Wae Mbeleng Health Center to wait for the research assistant and other health workers who brought the questionnaire sheets that had been filled out by the respondents. Generally, the assistants and health workers returned from the integrated health post and returned to the Health Center at 13.00 PM.

However, there were also those who arrived at the Health Center at 15.00 PM, depending on the distance and weather. Every day the researcher collected 8–10 questionnaires. There were also questionnaires that were not filled out and returned to the researcher because the target respondents did not come to the integrated health post. Then the questionnaires were distributed again to the research assistants and health workers to be taken to other integrated health post the next day. In cases where target respondents failed to attend the integrated health post, the researcher had encountered this challenge firsthand at the integrated health post of Lalang. Because the target respondent’s house was close to the integrated health post, the researcher visited the house and asked the respondent to fill it in and the data was collected at that time.

Another case was also found by health workers, that when the person who came to the integrated health post was a father who brought his child, the father was asked to fill out a questionnaire. The reason was that the father was the respondent’s husband who knew the respondent’s demographic data and knew the knowledge, attitudes and behavior of the respondent’s care for their stunted children. All questionnaires distributed by researchers to research assistants and health workers to be brought to each integrated health post every day, none were held back, all were returned on the same day, that day. Of the 26 integrated health posts visited, the target respondents of 56 respondents were only obtained at 20 integrated health posts for the reasons mentioned above.

### Data analysis

Data analysis in this study used the SPSS version 25 application to analyze univariate and bivariate data. The use of AI is limited to journal searches, not data analysis. Univariate analysis was used to see the frequency distribution of the description of the level of maternal knowledge and maternal attitudes in caring for stunted children aged 24–35 months. The knowledge variable is classified into three categories. Good knowledge if the score range is 7–10, sufficient knowledge if the score range is 4–6 and poor knowledge if the score range is 0–3. The attitude variable is classified into two categories. A positive attitude is said if the score range is > 61% and a negative attitude is said if the score range is ≤ 61%. The behavioral variable for caring for stunted children aged 24–35 months is classified into three categories. Good behavior is said if the score range is 121–180, sufficient behavior is said if the score range is 61–120 and poor behavior is said if the score range is 1–60.

Bivariate analysis was conducted to test the hypothesis whether there is a relationship between knowledge and attitudes with maternal behavior in caring for stunted children aged 24–35 months. In addition, to determine the relationship between education, maternal age, maternal occupation, family income or earnings, and the role of health workers in providing health education with maternal behavior in caring for stunted children aged 24–35 months. This study used parametric and non-parametric tests. The method used is Pearson Product Moment/Rank Spearman which aims to measure the correlation between two variables that are categorical and numerical or both numerical. Previously, a data normality test was conducted using Kolmogorov Smirnov because the sample size was ≥ 50. The independent variable (X1) whose data distribution was not normal was the knowledge variable (p = 0.000 < 0.05), while the independent variables (X2) and dependent variables (Y) whose data distribution was normal were the attitude and behavior variables in caring for stunted children aged 24–35 months which had the same significance value (p = 0.200 > 0.05). The significant value used is p > α = 0.05.

## Results

The results of the study on the sociodemographic characteristics of respondents can be seen in Table 1.

**Table 1:** Distribution of Respondent Characteristics Based on Demographic Data (n = 56).

Respondent characteristics	Categories	f	%
Mother’s education	Basic Education (Elementary School-Junior High School)	36	64.3
	Secondary Education (Senior High School)	13	23,2
	Higher education (Diploma-Doctoral)	7	12,5
Mother’s age	17-25 of age	6	10,8
	26-35 of age	32	57,1
	36-45 of age	18	32,1
Mother’s occupation	Working outside the home	41	73,2
	Working inside the home	15	26,8
	Not working	0	0
Family income	<2,1 million IDR	47	83.9
	>2,1 million IDR	9	16.1
Health counseling	Received	52	93
	Not received	4	7

Based on the table above, the majority of respondents are in early adulthood, 47 people (84%) and the average age is 32.50 years. For education, the majority of respondents have basic education (elementary school-junior high school) as many as 36 respondents (64.3%). For the respondents’ occupation, the majority work outside the home as many as 41 people (73.2%). For family work, the majority of respondents have an income below 2.1 million as many as 47 respondents (83.9%), while for respondents who received health counseling from health workers, all 56 people (100%) received counseling from health workers.

Table 2 Description of Mothers’ Knowledge about Stunted Child Care Behavior Aged 24-35 Months (n = 56) can be seen in Table 2.

**Table 2:** Description of Mothers’ Knowledge about Stunted Child Care Behavior Aged 24-35 Months (n = 56).

Variable of Knowledge	Median (min-max)	f	%
Mother’s knowledge about stunted child-care behavior aged 24–35 months	9 (7-10)	56	100

Description: n: Number of samples, f: frequency, %: percentage, p: significance > 0.05 data normality test using Kolmogorov Smirnov.

The table above shows that the median of mothers’ knowledge about caring behavior for stunted children aged 24–35 months is 9 with the lowest score being 7 and the highest score being 10. Table 3.

**Table 3:** Description of Mothers’ Attitudes about the Behavior of Caring for Stunted Children Aged 24-35 Months (n = 56).

Mother’s Attitude	Mean (±SD)	f	%
Mothers’ attitudes about the behavior of caring for stunted children aged 24–35 months	64, 96 (5,309)	56	100

The table above shows that all 56 respondents (100%) categorized mothers’ attitudes toward caring for stunted children aged 24–35 months as positive. The mean score for mothers’ attitudes toward caring for stunted children aged 24–35 months was 64.96, with a standard deviation of 5.309.

To test the relationship between mothers’ knowledge about the behavior of caring for stunted children aged 24–35 months, researchers used Pearson Product Moment statistics. Pearson product moment statistics are correlation tests that measure the closeness of the relationship between 2 independent variables and 1 dependent variable with an interval/ratio scale and return a coefficient value [30]. Table 4.

**Table 4:** Respondent Data Distribution Test Using the Kolmogorov-Smirnov Test.

No.	Variable Distribution	*p* (significance)	Conclusion
1.	Knowledge	0,000	Not normal
2.	Attitude	0,200	Normal
3.	Behavior	0,200	Normal

Description: Normality test using Kolmogorov-Smirnov (p > 0.05).

Based on the results of the variable distribution test above, it is known that the significance value of the knowledge variable (p = 0.000) < 0.05, which means that the data is not normally distributed. While in the attitude and behavior variables for caring for short children aged 24–35 months, each significance value is (p = 0.200) > 0.05, which means that the data is normally distributed. To find out the closeness of the relationship between variables, see Table 5.

**Table 5:** Relationship between Knowledge and Attitude Variables with Mother’s Behavior in Caring for Stunting Children Aged 24-35 Months Using the Pearson Product Moment and Spearman Rank Statistical Tests (n = 56).

No.	External Variable	Mother’s Behavior	
**r**	
**1.**	**Mother’s knowledge**	**-0,151**	**0,268** ^**a**^
**2.**	**Mother’s attitude**	**0,458**	**0,000** ^**b**^

Description: n: Number of samples; p: significance < 0.05: significant difference; a: Spearman’s Rank; b: Pearson Product Moment.

Based on the results of data processing on the knowledge variable using Spearman rank data analysis, it is known that the 2-tailed significance value of the knowledge variable (0.268) > 0.05 which concludes that the knowledge variable does not have a significant relationship with the variable of stunted child care behavior aged 24–35 months. While the results of data processing on the attitude variable using Pearson product moment data analysis with a 2-tailed significance value of the attitude variable (0.000) < 0.05 which concludes that the attitude variable has a significant relationship with the variable of stunted childcare behavior aged 24–35 months. To assess the closeness of the relationship between variables, the criteria for testing the Spearman rank correlation test and Pearson product moment must first be known.

From the table above, the correlation coefficient value of the knowledge variable at r count of -0.151 means that the direction of the relationship between the two variables is negative/not in the same direction, which means that if knowledge increases, then the behavior of caring for stunted children aged 24–35 months decreases, or if knowledge decreases, then the behavior of caring for stunted children aged 24–35 months increases. While the correlation coefficient value of the attitude variable in the calculated r above is 0.458, meaning that it has a positive/unidirectional relationship, which means that if the attitude variable increases, the behavior of caring for stunted children aged 24–35 months will also increase, or if the attitude decreases, the behavior of caring for stunted children aged 24–35 months will also decrease. To find out the correlation value and level or strength of the relationship in the Spearman rank correlation test and Pearson product moment, it can be seen in Table 6.

**Table 6:** Criteria for Testing Spearman Rank Correlation and Pearson Product Moment Based on Correlation Interpretation.

No.	Correlation Value	Level of Significance
1.	0,00-0,199	Very weak
2.	0,20-0,399	Weak
3.	0,40-0,599	Medium/sufficient
4.	0,60-0,799	Strong
5.	0,80-1,00	Very strong

From the r table above, the knowledge value in the calculated r is -0.151, between 0.00-0.199 which is significant, the strength/level of the relationship between the knowledge variable and the behavior of caring for stunted children aged 24–35 months is very weak. While the calculated r value on the attitude variable is 0.458, between 0.40-0.599 which is significant, the strength of the relationship between the attitude variable and the behavior of caring for stunted children aged 24–35 months is classified as moderate. To test the relationship between external variables and maternal behavior in caring for stunted children aged 24–35 months, the researcher used Spearman Rank and Pearson Product Moment statistics with the results that can be seen in Table 7.

**Table 7:** Relationship between External Variables and Mother’s Behavior in Caring for Stunted Children Aged 24-35 Months Using Spearman Rank and Pearson Product Moment Statistical Tests (n = 56).

No.	External Variable	Mother’s Behavior	
		r	p
**1.**	Mother’s Education	0,269	0,045^a^
**2.**	Mother’s Age	-0,365	0,006^b^
**3.**	Mother’s Occupation	0,080	0,558^a^
**4.**	Family Income	0,202	0,136^a^
**5.**	Health Counseling	0,109	0,422^a^

Description: n: Number of samples; p: significance < 0.05: significantly different; a: Spearman’s Power; b: Pearson Product Moment.

Based on the results of data processing from the table above, it is known that the significance value of the 2-tailed variable of maternal education (0.045) <0.05 which concludes that the variable of maternal education has a significant relationship with the variable of behavior in caring for stunted children aged 24–35 months.

Based on the results of data processing from the table above, it is known that the significance value of the 2-tailed variable of maternal age (0.006) <0.05 which concludes that the variable of maternal age has a significant relationship with the variable of behavior in caring for stunted children aged 24–35 months.

Based on the results of data processing from the table above, it is known that the significance value of the 2-tailed variable of maternal occupation (0.558) > 0.05 which concludes that the variable of maternal occupation does not have a significant relationship with the variable of behavior in caring for stunted children aged 24–35 months. Based on the results of data processing from the table above, it is known that the significance value of the 2-tailed family income variable (0.136) > 0.05 which concludes that the family income variable does not have a significant relationship with the behavioral variable of caring for stunted children aged 24–35 months.

Based on the results of data processing from the table above, it is known that the significance value of the 2-tailed health worker counseling variable (0.422) > 0.05 which concludes that the health worker counseling variable does not have a significant relationship with the behavioral variable of caring for stunted children aged 24–35 months.

## Discussion

The data shows a significance value of knowledge p = 0.268 > 0.05 and r = -0.151. This concludes that the knowledge variable has a negative/insignificant relationship with the variable of childcare behavior for stunting aged 24–35 months. A study in Ethiopia contradicts the findings of researchers, that mothers who are well-informed about optimal childcare behaviors (complementary feeding practices) tend to have better complementary feeding practices compared to mothers with poor knowledge (p = 0.01 < 0.05) [31].

Another conflicting study was conducted at the Sekatak Buji Community Health Center in North Kalimantan. The chi-square test showed a p-value of 0.000 < 0.05. Thus, the prior knowledge variable significantly impacted the care behavior variable (feeding behavior domain). This positively indicates that when mothers actively seek information and knowledge about providing complementary foods correctly, they will practice good and correct complementary feeding behaviors for their children [32]. Because the formation of behavior is an evolution of knowledge that can shape attitudes and subsequently have an impact on the formation of behavior, knowledge about caring for stunted children (feeding behavior in children) can influence the behavior of mothers in feeding their children [33].

The findings of this study show a significant Favalue of attitude p = 0.000 < 0.05 and r = -0.458. This concludes that the attitude variable has a positive/significant relationship with the behavioral variable of caring for stunted children aged 24–35 months. A study in Ethiopia supports this finding, that mothers who have a positive attitude towards optimal childcare behavior (complementary feeding practices) tend to have better complementary feeding practices compared to mothers who have a negative attitude (p = 0.01 < 0.05) [31].

Another study at the P-S Health Center also supports the researcher’s findings that there is a significant relationship between maternal attitudes and patterns of childcare behavior (feeding behavior domain) aged 12–24 months with a significance value of p = 0.004 < 0.05 and a prevalence ratio value of 1.19. This shows that maternal attitudes have a positive relationship with childcare behavior aged 12–24 months, namely in the domain of feeding behavior.

Thus, mothers who have less attitudes are less likely to behave poorly in feeding children aged 12–24 months. Conversely, mothers who have good attitudes regarding proper feeding of children will be directly proportional to their behavior [34].

Based on the bivariate analysis between the level of maternal education and maternal behavior in caring for stunted children aged 24–35 months, the significance value is p = 0.045 < 0.05. The same study also occurred in all South Asian countries that maternal education has a significant and consistent positive relationship with childcare behavior (feeding practice domain). This relationship has an important role in improving child feeding practices [35].

Based on the bivariate analysis between maternal age and maternal behavior in caring for stunted children aged 24–35 months, the significance value (p = 0.006) <0.05. Supporting research occurred in Cameroon with a significant value of maternal age related to nutritional status (feeding behavior domain) of stunted children aged 5–24 months (p = 0.006 < 0.05) [36].

Based on bivariate analysis between maternal occupation and maternal behavior in caring for stunted children aged 24–35 months, the significance value (p = 0.558) > 0.05. Another study in Eastern Ethiopia explained the opposite, that stunting in children occurs due to poor feeding behavior which is significantly related to maternal employment status (p < 0.05). This results in a 1.71 times higher risk of stunting in children born to mothers who work outside the home compared to children born to mothers who work at home/housewives with AOR = 1.71; 95% CI: 1.08–2.72 [37]. Based on the results of interviews with several mothers at the research site, the thing that supports the reduction in stunted child care behavior aged 24–35 months is that mothers in Manggarai (Wae Mbeleng Health Center) who work as farmers work more in the fields than men (husbands), so that their energy is divided between working outside the home (farming) and working at home as housewives in caring for stunted children aged 24–35 months. This reason is one indicator that other factors such as culture can be an obstacle to caring for stunted children aged 24–35 months at the Wae Mbeleng Health Center and can also be used as a basis for further research. The results of the bivariate test between family income and maternal behavior in caring for stunted children aged 24–35 months, significance value (p = 0.136) > 0.05. Different studies are in Dhaka, Bangladesh with the significance value of monthly family income with nutritional status of stunted children < 2 years (childcare behavior in the domain of feeding) is p = 0.02 < 0.05 [38]. Family income below the minimum wage can reduce food purchasing power and can further inhibit the behavior of caring for stunted children aged 24–35 months. Based on the bivariate analysis between the provision of health education to mothers and the behavior of mothers in caring for stunted children aged 24–35 months, the significance value is p (0.422) > 0.05. Different studies in India show that there is a significant relationship between mothers and stunted children aged 6–23 months who receive counseling on the behavior of care in the domain of feeding (nutrition in antenatal care) with determinants of stunting. AOR value 1.318 [39,40]. Based on the study’s findings, the insignificant relationship may be attributed to the influence of other factors, such as the participants’ level of knowledge being limited to understanding, without reaching the higher levels of appreciation, internalization, and implementation. The transformation and optimization of knowledge about stunted childcare obtained from health workers requires strong awareness and commitment to its importance in improving the care, growth, and development of children aged 24–35 months.

### Strengths and limitations

A key strength of this study is its focus on a specific target population, namely mothers of children aged 24–35 months with stunting. The second strength lies in the use of four domains of stunted childcare behavior instruments (feeding behavior domain, psychosocial stimulation domain, environmental sanitation behavior domain, and treatment seeking domain for sick children). The limitations of this study lie in the relatively small sample size, which can limit the generalization of the findings to a wider population. The solution offered is to suggest that further researchers look for larger and wider research samples. Another limitation is that the references to this journal are mostly found in the researcher’s country, making it difficult to find differences and similarities from other developing countries affected by stunting. The solution is to strengthen research collaboration with foreign authors or institutions and try to subscribe to paid access journals.

## Conclusion

There is a significant relationship between the frequency distribution of maternal education (p = 0.045 < 0.05), maternal age (p = 0.006 < 0.05) with maternal behavior in caring for stunted children aged 24–35 months. There is no significant relationship between the frequency distribution of maternal employment (p = 0.558 > 0.05), family income (p = 0.136 > 0.05), and health education from health workers (p = 0.422 > 0.05) with maternal behavior in caring for stunted children aged 24–35 months. The results of the bivariate analysis showed that the significance value of the 2-tailed knowledge variable with maternal behavior in caring for stunted children aged 24–35 months was not in the same direction/negative (p = 0.268) > 0.05. This concludes that the maternal knowledge variable does not have a significant relationship with the maternal behavior variable in caring for stunted children aged 24–35 months. The results of the bivariate analysis showed that the 2-tailed significance value of the attitude variable (p = 0.000) < 0.05, which concluded that the attitude variable had a positive relationship with the behavioral variable of maternal care for stunted children aged 24–35 months.
